# Accelerated wound healing and reduced scar formation induced by D-mannose: a possible role of mannose binding lectin

**DOI:** 10.1007/s00403-024-03338-w

**Published:** 2024-09-03

**Authors:** Cristian Ionuț Ciucanu, Sonia Rațiu, Gianina Elena Crîșmariu, Sorin Olariu, Ionel Ciucanu

**Affiliations:** 1 Faculty of Medicine, University of Medicine and Pharmacy “Victor Babes” of Timişoara, Piaţa Eftimie Murgu 2, 300041 Timişoara, Romania; 2 Department of General Surgery I, County Emergency Hospital „Pius Brinzeu” Timisoara, Timisoara, Romania; 3 Faculty of Medicine and Pharmacy, Oradea University, Oradea, Romania; 4 Faculty of Chemistry, Biology, Geography, West University of Timisoara, Timișoara, Romania

**Keywords:** Wound healing, Surgery, Repair of damaged tissue, Mannose, Wound infection

## Abstract

Wounds are most commonly caused by accidents, surgery and burns, and can be internal or external. Naturally, the wound healing process can take a long time and lead to scarring. In this study we present a technique to shorten wound healing time and prevent or mitigate scarring using D-mannose that is applied directly on the wound. The results showed that the healing time is almost halved compared to treatment with povidone-iodine solution which is an antiseptic widely used in surgery. D-Mannose is neither an antisepsis nor an antibiotic. We propose a posssible mechanism by which D-mannose binds to D-mannose binding lectin and immediately activates the innate immune system that ultimately phagocytizes pathogens and clears the wound of degraded cells and materials, which reduces inflammation and implicitly wound healing time. D-Mannose also intervenes in the coagulation process by binding to fibrinogen, generating a finer and denser fibrin, which visibly reduces collagen scars. Our findings show that applying D-mannose directly on the wound as a powder shortens wound healing time and visibly attenuates scarring. Apart from the unaesthetic appearance, these scars can also cause a certain tissue dysfunction, regardless of the affected organ.

**Graphical abstract:**

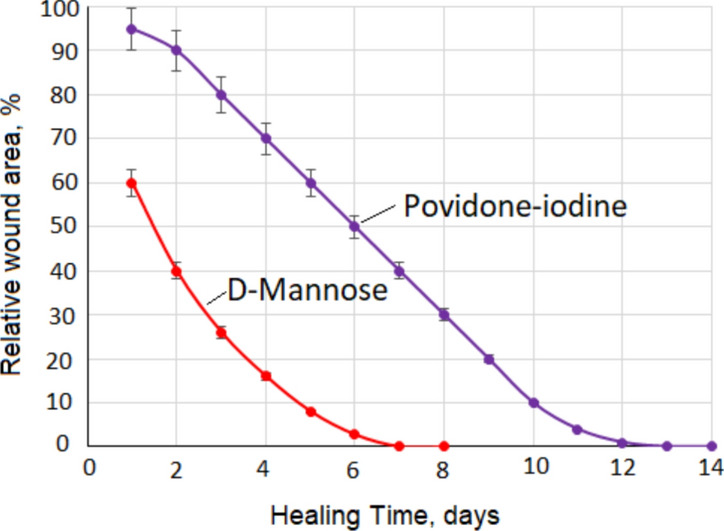

## Introduction

When tissue is injured, the nervous system activates the stress response by sending impulses from the injured site to the hypothalamus [1]. These impulses will generate a cascade of changes in metabolic and physiological processes to restore homeostasis. As a result, changes in inflammatory and hormonal responses will be induced to destroy infectious organisms, limit tissue damage and activate the repair processes of damaged tissues. The global metabolic effect of the endocrine response is the mobilization of carbohydrates, lipids, and proteins from stores.

Wounded tissue healing is a natural process that consists of a series of dynamic processes, beginning with hemostasis and the inflammatory process, continuing with tissue formation, and ending with remodeling of damaged tissues [2]. Hemostasis is marked by platelet accumulation, coagulation and leukocyte migration and lasts a few minutes. Simultaneously with coagulation, the inflammatory process and the immune system are activated in order to prevent wound infection. Tissue formation is characterized by reepithelialization, angiogenesis, fibroplasia, and wound contraction and lasts up to 14 days [2]. Finally, the remodeling phase occurs over a period of several months, during which the dermis responds to the injury with the production of collagen and matrix proteins and then returns to its pre-injury phenotype [3]. However, collagen deposits in the wound will lead to some tissue dysfunction, regardless of the affected organ. That is why it is desired that the collagen deposits in the scar be as small as possible.

The time interval for restoring damaged tissue is quite long and depends on the microenvironment in which this complex repair process takes place. The biggest danger is wound infection with various pathogens. Wound infections can be in superficial incisions, in deep incisions and infections of body organs.

In addition to antiseptics and antibiotics known and used to avoid wound infection, numerous studies have shown that many human wounds can be successfully treated since ancient times by applying on the wound sugar granules [4], honey [5] or Aloe Vera juice [6]. Studies [7] made with solutions of glucose, fructose, galactose, and D-mannose injected subcutaneously in mice, showed that glucose and fructose have no effect on the formation of granulation tissue, galactose increased the amount of granulation tissue, and D-mannose decreased amount of granulation tissue. Another study [8] carried out on mice shows that subcutaneous injection of D-mannose leads to the inhibition of hyaluronan synthesis, while glucose, fructose, and galactose do not produce this effect. The authors say that this effect could explain a decrease in granulation tissue after injection with D-mannose solution.

A series of polymers that have glucose and D-mannose in their structure have also been used for wound healing [9]. A patent was also published for treating wounds with a D-mannose-6-phosphate solution [10]. There is no study on wound healing by direct application of D-mannose as a powder or aqueous solution on the wound surface.

The aim of this study is to present for the first time the wound healing acceleration effect that D-mannose induces by applying D-mannose directly to the wound in the form of powder, as well as the possible role of mannose-binding lectin (MBL) in this process.

## Materials and methods

### Materials

D-Mannose powder of microbiological purity was from Merck (Darmstadt, Germany). The 10% povidone-iodine solution, was from Egis Pharmaceuticals, Hungary. The adhesive tapes and sterile compresses and dressings were from Hartmann, Romania. Medical distilled water was from Braun, Germany.

### Subjects

Eight Caucasian men over the age of 50 participated in this study. Subjects with any form of cardiovascular disease, diabetes, kidney or liver disease, thyroid disease, autoimmune disorders, smoking, and inflammatory diseases were excluded. Subjects did not take any medication during the study period. All followed their usual diet throughout the study. They completed a personal medical history questionnaire and a complete physical examination.

### Study design and methods

This study was a randomized, double-blind, crossover experiment involving a single group of subjects. In an upper area of the front part of the thigh of each subject were made 2 incisions of 4 mm using a surgical knife. One incision per day was made on each volunteer. Each area was first sterilized according to the surgical protocol and then locally anesthetized with 1–2 puffs of kellen spray. The incision involved the skin and subcutaneous tissue. Immediately after the incision, each wound was opened and the treatment procedure followed. Each wound was subjected to a single treatment. Treatments were randomized so that at the end, each subject was treated with powder of D-mannose and povidone-iodine solution. D-mannose powder and povidone-iodine solution was applied so as to completely cover the wound. All wounds were covered with sterile non-adhesive dressings. Either D-mannose or povidone-iodine was applied to the dressings. Wound dressings were immobilized with elastic adhesive bandages. The wounds were not sutured. For the next 7 days, this procedure was repeated every day after the wounds were cleaned with medical distilled water. After this period, the wounds were protected only with a sterile dressing. Wounds were photographed and wound dimensions were measured. No antibiotics or anticoagulants were administered during the study.

### Wound evaluation

The wound healing evaluation was photographed with a digital camera A715 Samsung, South Korea with 8 magnifications (3468 × 4624 pixels). The relative wound area of all wounds was calculated using the following equation:

1 $${\text{Relative wound area }}\left( \% \right) = \left( {{\text{Ai}}/{\text{Ao}}} \right) \, \times { 1}00,$$

where Ai and Ao represent the intermediate wound area and initial wound area, respectively.

The area of the wound was approximated by the area of an ellipse and therefore the length and width of the wound were measured daily with the aim of being entered into the formula of the area of the ellipse.

### Statistical analyses

In this study, the change over time in the relative area of two wounds on each subject in a group of eight subjects was followed. One wound was treated with D-mannose and another wound with povidone-iodine solution. This variable is expressed numerically as mean ± standard deviation (SD). All statistical analyzes were performed with the Statistical Test Calculator online version 2018 from Social Science Statistics (https://www.socscistatistics.com/tests/) and Microsoft Excel 2016 (Microsoft Corp. USA).

## Results and discussion

**Fig. 1:**
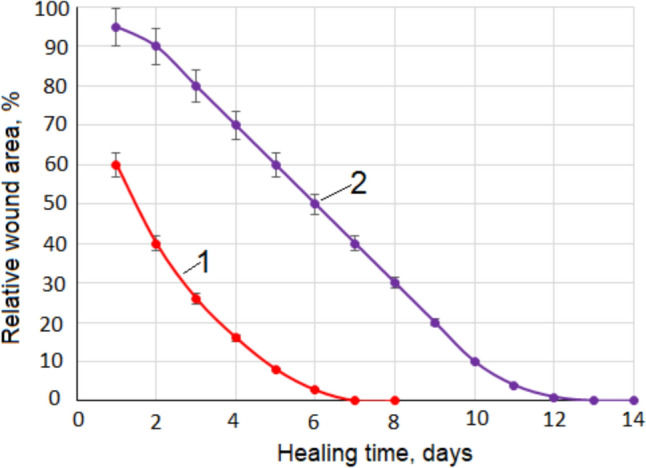
Variation of relative wound area as a function of time. **1**—Treating the wound with D-mannose powder. **2**—Treating the wound with povidone-iodine solution

Figure 1 shows how the relative wound area changed after applying D-mannose powder or povidone-iodine solution to the wound. The wound healing effect of D-mannose was compared with povidone-iodine solution. This effect was followed by daily measurement of wound width and length. The graph represents the percentage change over time in the area of the wound relative to the initial area. It can be seen from Fig. 1 that the wound healing time with D-mannose is almost half of the healing time with povidone-iodine solution. From Fig. 1, it can be seen that the percentage variation of the relative wound area, shown on the Y-axis, as a function of the number of healing days, shown on the X-axis, is not linear. Nonlinear functions fit practical data. For D-mannose it was a polynomial equation of the second degree, Y = 1.51X^2^—21.91X + 79.14 (R^2^ = 0.9983), for X ∈ [1, 8], and for the povidone-iodine solution it was a polynomial equation of the third degree, Y = 0.0704X^3^ – 1.2549X^2^ – 3.3382X + 99.865 (R^2^ = 0.9994), for X ∈ [1, 14]. R^2^ is the coefficient of determination. These equations describe very well the variation of the relative wound area as a function of the healing time in the indicated domains, because the closer R^2^ is to the value 1 the better the function matches the experimental values.

**Fig. 2:**
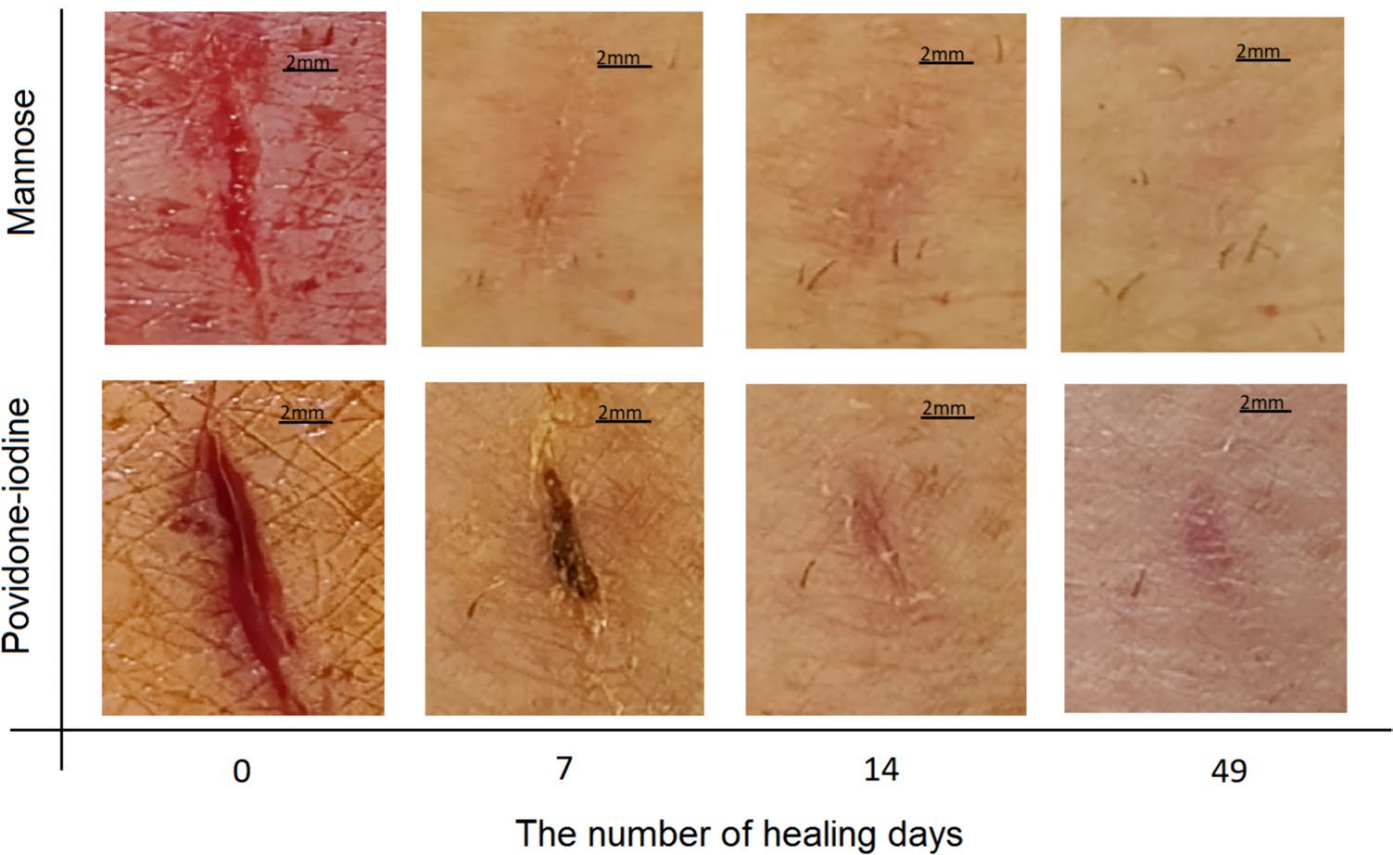
Visual observation over time of the degree of wound healing

Figure 2 contains 8 images magnified 8 times showing the wound on the day of the incision and after 7, 14 and 49 days of healing, respectively. Four images are for treatment with D-mannose and in comparison are 4 images for treating the wound with povidone-iodine solution. It was noted that when D-mannose is applied on the wound, the stinging sensation does not appear. On the other hand, when treated with povidone-iodine solution, there is a slight stinging on the first and second day. With D-mannose, it was observed that the dressing applied on the wound did not stick on the wound the next day and was easily removed. With the povidone-iodine solution, the dressing was adhesive to the wound the next day, resulting in slight destruction of the granulation tissue when it was removed. With the povidone-iodine solution, reddening of the skin around the wound was observed during the first 4 days of healing, indicating mild inflammation. With D-mannose, this redness disappears after the first day. After 7 days, the wounds treated with D-mannose and respectively after 14 days, the wounds treated with povidone-iodine solution are closed and completely epithelized. After 49 days, wounds treated with povidone-iodine solution show a scar at the site of the wound. By comparison, healing with D-mannose treatment was very good after 49 days and no visible signs of scarring.

D-mannose is a monosaccharide present in both the plant and animal kingdoms. In the blood, D-mannose is also found in the free state in concentrations tens of times lower than glucose and is determined by chromatographic methods [11, 12]. Being present in many foods and in the blood, D-mannose has not been shown to be a toxic substance for the human body. In addition, D-mannose can interconvert in the human body into glucose and fructose [13]. In the human body, D-mannose is mainly in the structure of some glycoproteins and glycolipids, which are obtained in the liver by glycosylation with D-mannose.

When a tissue is injured, the body triggers hemostasis and an inflammatory process to stop bleeding through broken blood vessels, clear damaged cells and tissues, initiate tissue repair, and prevent infection of the wound by external infectious agents. Although the inflammatory process is a necessary physiological process for wound healing, external pathogens induce excessive inflammation that prolongs healing time.

There are many antiseptics for killing pathogens, but only a few are relevant in treating wound infections [14]. Among them, povidone-iodine solution has a great application in surgery, and therefore we chose it as a comparison element for the effect of D-mannose. The antimicrobial action of the povidone-iodine solution is manifested by inhibiting the vital processes of pathogens due to the oxidizing properties of iodine [14].

Several studies have shown that D-mannose also has positive effects in treating some bacterial infections in the kidneys, later demonstrating that it can reduce inflammation in many diseases [13, 15]. D-Mannose is not an antibiotic or antiseptic because it does not act directly on infectious agents. The action is more complicated in the case of wound healing.

We propose in this study a possible mechanism by which D-mannose acts in wound healing. This mechanism primarily assumes the involvement of mannose-binding lectin (MBL), which is a protein of the innate immune system. MBL is a protein synthesized in the liver that is found in plasma and has the ability to recognize carbohydrates with which pathogens bind to host cells.

In the first moments after injury, the body's response to pathogens is through an innate immune system activated by MBL. The complement system in the immune system is activated when MBL binds to a pathogen-specific monosaccharide such as D-mannose. A complex cascade process follows that ultimately determines the phagocytosis of a large number of pathogens by macrophages and neutrophils [16]. Through this mechanism, MBL will be activated by the high concentration of D-mannose applied to the wound and will act through the innate immune system to protect the wound from pathogens, reducing the time required for wound healing.

**Fig. 3:**
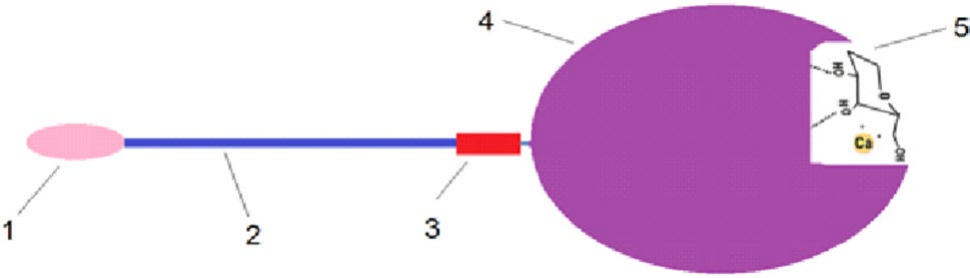
Structure of MBL complexed with a D-mannose molecule. **1**—Cysteine-rich domain. **2**—Collagen-like region. **3**—Neck region. **4**—Carbohydrate-recognition domain. **5**—D-mannose molecule

MBL is a complex structure of protein subunits linked as a bundle [16]. Each protein subunit consists of a cysteine-rich domain, a collagen-like region, a neck region, and a carbohydrate-recognition domain. Figure 3 shows the component parts of a subunit of MBL and the complex it forms with D-mannose. The carbohydrate recognition domain contains receptors that form a calcium ion (Ca^2+^)-dependent complex with the monocarbohydrate through hydrogen bonds established with equatorially oriented hydroxyl groups in positions 3 and 4 of the monocarbohydrate molecule [17]. This explains the high specificity of MBL for D-mannose molecule, which has such a chemical structure.

This system is designed so that MBL recognizes only pathogens that have D-mannose molecules on their surface that are not embedded in a polymer structure, but are terminal molecules of various polymer branches [15].

By adding D-mannose to the wound, it will quickly dissolve into the plasma. The added D-mannose is thus free and will bind to the receptor in the MBL, which will rapidly activate the immune system that will ultimately stimulate phagocytes to clear the wound of decayed cells and materials that are foreign to the body. Through this mechanism it is possible to reduce the inflammatory reaction, which will lead to a decrease in the time required for wound healing, as can be seen in Fig. 1.

The fact that applying povidone-iodine to the wound leads to an increase in healing time compared to D-mannose is partly due to the fact that iodine is a non-selective oxidant and destroys by oxidation all cells it enters. As a result, inflammation will increase to remove these damaged cells. Increased inflammation is indicated by reddened skin around the wound in the first days after treatment. This increase in inflammation explains why the healing time was longer when we applied povidone-iodine solution directly to the wound.

In addition, by applying the povidone-iodine solution, a lot of foreign chemical compounds are introduced into the wound with this solution, which must be removed from the wound, and this process also causes an increase in inflammation. Instead, D-Mannose is a compound that occurs naturally in the bloodstream and does not cause degradation in the body, nor does it bring unknown compounds to the body.

A recent study [18] on fibrinogen showed that some carbohydrates and glycoconjugates can influence the formation of fibrin clots. The authors of the study show that fibrinogen has specific receptors that can fix monocarbohydrates that influence the polymerization process to fibrin. Thus, in the presence of methyl-α-D-mannose, thinner and more branched fibrin fibers were obtained, which makes the fibrin polymer finer and more resistant. Also, another study [7] concluded that D-mannose injected subcutaneously decreases the excessive production of extracellular material that is deposited in the wound matrix.

These studies may explain why the D-mannose treatment we applied to the wound resulted in scarring that was practically unnoticeable compared to the povidone-iodine solution treatment. Wound scarring was observed with povidone-iodine treatment, as large amounts of extracellular material and collagen are deposited in the wound matrix, as can be seen in Fig. 2 on the seventh day of healing. This extracellular material is difficult to remove by specific enzymes such as collagenase. Apart from the unaesthetic appearance, these scars can also cause a certain tissue dysfunction, regardless of the affected organ.

In a future study we propose to analyze the effect of D-mannose on wound healing in the case of elderly patients who use drugs such as rosuvastatin [19] with a strong impact in preventing conditions caused by atherosclerosis [20].

This study may have some limitations given the relatively small number of male subjects and a small age range. The first limitation was reduced by using a crossover experiment [21] with two treatments and a homogeneous group of Caucasian men. Future studies should include a larger group of subjects, such as women and other age groups, as well as other compounds for comparison [22], so that there can be more extensive validation of the results. This study should be viewed with these limitations.

## Conclusions

The primary medical objective of this study was to reduce wound healing time and prevent or mitigate scarring. The effect on wound healing time was observed by applying D-mannose directly to the wound. The results showed that the healing time by applying D-mannose directly to the wound is almost halved compared to treating the wound with povidone-iodine solution. When the wound was treated with D-mannose, the newly formed epithelium had no visible signs of collagen-induced scarring because D-mannose binds to fibrinogen and influences the formation of fine, branched fibrin fibers.

## Data Availability

All data generated or analyzed during this study are included in this article. Further enquiries can be directed to the corresponding author on reasonable request. No datasets were generated or analysed during the current study.

## References

[1] Finnerty CC, Mabvuure NT, Ali A, Kozar RA, Herndon DN (2013) The surgically induced stress response. J Parenter Enteral Nutr 37:21S-29S. 10.1177/0148607113496117 10.1177/0148607113496117 PMC3920901 24009246

[2] Gurtner GC, Werner S, Barrandon Y, Longaker MT (2008) Wound repair and regeneration. Nature 453:314–321. 10.1038/nature07039 18480812 10.1038/nature07039

[3] Wu YS, Chen S-N (2014) Apoptotic cell: linkage of inflammation and wound healing. Front Pharmacol 5:1. 10.3389/fphar.2014.00001 24478702 10.3389/fphar.2014.00001 PMC3896898

[4] Knutson RA, Merbitz LA, Creekmore MA, Snipes HG (1981) Use of sugar and povidone-iodine to enhance wound healing: five years’ experience. South Med J 74:1329–1335. 10.1097/00007611-198111000-00010 7302631 10.1097/00007611-198111000-00010

[5] Efem SEE (1988) Clinical observations on the wound healing properties of honey. Br J Surg 75:679–681. 10.1002/bjs.1800750718 3416123 10.1002/bjs.1800750718

[6] Zeng WM, Parus A, Barnes CW, Hiro ME, Robson MC, Payne WG (2020) Aloe vera—mechanisms of action, uses, and potential uses in plastic surgery and wound healing. Surg Sci 11:312–328. 10.4236/ss.2020.1110033

[7] Kössia J, Peltonenb J, Ekforsc T, Niinikoskia J, Laatoa M (1999) Effects of hexose sugars: glucose, fructose, galactose and mannose on wound healing in the rat. Eur Surg Res 31:74–82. 10.1159/000008623 10072613 10.1159/000008623

[8] Jokela TA, Kuokkanen J, Kärnä R, Pasonen-Seppänen S, Rilla K, Kössi J et al (2013) Mannose reduces hyaluronan and leukocytes in wound granulation tissue and inhibits migration and hyaluronan-dependent monocyte binding. Wound Repair Regen 21:247–255. 10.1111/wrr.12022 23464634 10.1111/wrr.12022

[9] Wong CCQ, Tomura K, Yamamoto O (2023) Wound healing performance in a moist environment of crystalline glucose/mannose film as a new dressing material using a rat model: comparing with medical-grade wound dressing and alginate. Pharmaceuticals 16:1532. 10.3390/ph16111532 38004398 10.3390/ph16111532 PMC10674295

[10] Ferguson MWJ (2000) Mannose-6-phosphate composition and its use in treating fibrotic disorders. US Pat. 6093388

[11] Campi B, Codini S, Bisoli N, Baldi S, Zucchi R, Ferrannini E et al (2019) Quantification of d-mannose in plasma: Development and validation of a reliable and accurate HPLC-MS-MS method. Clin Chim Acta 493:31–35. 10.1016/j.cca.2019.02.024 30802440 10.1016/j.cca.2019.02.024

[12] Ciucanu I, Pilat L, Ciucanu CI, Şişu E, Dumitraşcu V (2016) Simultaneous analysis of neutral monosaccharides, fatty acids and cholesterol as biomarkers from a drop of blood. Bioanalysis 8:2147–2156. 10.4155/bio-2016-0107 27611641 10.4155/bio-2016-0107

[13] Sharma V, Ichikawa M, Freeze HH (2014) Mannose metabolism: more than meets the eye. Biochem Biophys Res Commun 453:220–228. 10.1016/j.bbrc.2014.06.021 24931670 10.1016/j.bbrc.2014.06.021 PMC4252654

[14] Bigliardi PL, Alsagoff SAL, El-Kafrawi HY, Pyon J-K, Wa CTC, Villa MA (2017) Povidone iodine in wound healing: a review of current concepts and practices. Int Surg J 44:260–268. 10.1016/j.ijsu.2017.06.073 10.1016/j.ijsu.2017.06.073 28648795

[15] Dhanalakshmi M, Sruthi D, Jinuraj KR, Das K, Dave S, Andal NM et al (2023) Mannose: a potential saccharide candidate in disease management. Med Chem Res 32:391–408. 10.1007/s00044-023-03015-z 36694836 10.1007/s00044-023-03015-z PMC9852811

[16] Kalia N, Singh J, Kaur M (2021) The ambiguous role of mannose-binding lectin (MBL) in human immunity. Open Med 16:299–310. 10.1515/med-2021-0239 10.1515/med-2021-0239 PMC7917369 33681468

[17] Feinberg H, Castelli R, Drickamer K, Seeberger PH, Weis WI (2007) Multiple modes of binding enhance the affinity of dc-sign for high-mannose n-linked glycans found on viral glycoproteins. J Biol Chem 282:4202–4209. 10.1074/jbc.M609689200 17150970 10.1074/jbc.M609689200 PMC2277367

[18] Date K, Ohyama M, Ogawa H (2015) Carbohydrate-binding activities of coagulation factors fibrinogen and fibrin. Glycoconj J 32:385–392. 10.1007/s10719-015-9603-9 26050259 10.1007/s10719-015-9603-9

[19] Ciucanu CI, Ciucanu I, Olariu S (2022) Derivatization of rosuvastatin as methyl ester for its analysis by gas chromatography-mass spectrometry in plasma. Stud Univ Babes-Bolyai, Chem 67:19–26. 10.24193/subbchem.2022.4.02

[20] Ciucanu CI, Olariu S, Vlad DC, Dumitraşcu V (2020) Influence of rosuvastatin dose on total fatty acids and free fatty acids in plasma: correlations with lipids involved in cholesterol homeostasis. Medicine 99:e23356. 10.1097/MD.0000000000023356 33235104 10.1097/MD.0000000000023356 PMC7710209

[21] Jones B, Kenward MG (2014) Design and analysis of cross-over trials, 3rd edn. Chapman and Hall/CRC Press, New York

[22] Ciucanu CI, Ciucanu I (2024) Compositions and methods for rapid wound healing and reduction of scarring. RO Pat. Appl. No. A/00175

